# A Manual of Materia Medica

**Published:** 1839-10

**Authors:** 


### Art. II.

1. *Lehrbuch der Arzneimittellehre.* Von Dr. C. G. Mitscherlich. I. Theil. 1 Abth.—*Berlin*, 1837. 8vo, pp. 126.

*A Manual of Materia Medica.* By Dr. C. G. Mitscherlich. Part I. Division 1.—*Berlin*, 1837.

2. *The Elements of the Materia Medica; comprehending the Natural History, Preparation, Properties, Composition, Effects, and Uses of Medicines.* Part I., *containing the General Action and Classification of Medicines, and the Mineral Materia Medica.* By Jonathan Pereira, f.r.s., &c.—*London*, 1839. 8vo, pp. 559.

3. *Die Neuesten Entdeckungen in der Materia Medica. Für praktische Aerzte geordnet.* Von Dr. J. H. Dierbach. 2te Ausgabe. Ier Band.—*Heidelberg und Leipzig*, 1837. 8vo, pp. 656.

*The Latest Discoveries in the Materia Medica, arranged for Medical Practitioners.* By Dr. J. H. Dierbach. *2d Edition. 1st volume. Heidelberg and Leipsic*, 1837.

4. *Von dem Wirkungen der gebräuchlichen Metalle auf den menschlichen Organismus überhaupt, und als Heilmittel; und dem Kupfersalmiak Liquor, und andern Kupferpräparaten als solchen insbesondere.* Von Dr. J. R. Köchlin.—*Zürich*, 1837. 8vo, pp. 186.

*Of the Effects of the Metals in common use upon the Human Organism generally, and as Remedial Agents; and of the Cupro-Ammoniacal Liquor, and other preparations of Copper, as such, in particular.* By Dr. J. R. Köchlin.—*Zurich*, 1837.

5. *Physiologisch-therapeutische Untersuchungen über das Veratrin.* Von Dr. F. A. Forcke.—*Hannover*, 1837. 8vo, pp. 146.

*Physiological and Therapeutical Researches concerning Veratrine.* By Dr. F. A. Forcke.—*Hanover*, 1837.

I. Dr. Mitscherlich, favorably known as a scientific physician by his ingenious researches into chemical pathology, has presented us, in the present volume, with the first part of his work on materia medica and therapeutics. It embodies the most recent doctrines of modern physiology, and must be considered, as far as it goes, a meritorious performance.

After pointing out the influence of mind and various external agencies on the animal frame, the author proceeds to the principal subject of the work.

"The material remedies derived from the three kingdoms of nature are partly elementary combinations according to definite proportions, and consequently always uniform in composition; developing, under similar circumstances, the same phenomena in the economy, the result, as will be afterwards proved, of their chemical properties. Such, however, is not the case with a vast number of substances, the products of animal and vegetable life. It is true, indeed, that among these we still find definite elementary combination, as the alkaloids and acids, to which the same principle applies: but in others, where the proportions are not fixed, as roots, bark, wood, flowers, milk, musk, and the like, the phenomena are variable, in consequence of their chemical composition being modified by the age, influence of nutrition, light, and heat." (p. 9.)

The material remedies are divided, as usual, into solid, liquid, and aëriform. Of course, the author observes, many articles of diet play the part of medicinal agents, when it happens that a disease can be mitigated or removed by a change in the nutrition of the body.

The second section is devoted to the forms and modes of exhibiting medicines. In mentioning the pharmaceutical operations of pulverization and solution, we are told (p. 12) that "the *lac sulphuris*, an impalpable powder, acts much more powerfully than the *flores sulphuris*, which consist of fine crystals." Now we have ascertained by accurate microscopical observation the very converse to be the fact: the *lac sulphuris* consists of delicate crystals, while the *flores sulphuris* are drusy, globular masses, more or less coherent one to another. Indeed, by this same test, it will be seen that every precipitated powder is crystalline in structure.

In treating of the changes which a remedy undergoes in the living organism, the author discusses those which it suffers at the immediate point of contact, during absorption, and lastly during elimination by the different emunctories, as the kidneys, skin, lungs, &c. On this important head he admits that our knowledge is hitherto confined to very few remedies; but we are enabled to infer from analogy the effects of the rest.

"The decomposition of remedies at the first point of contact is always in accordance with the laws of chemistry and physics, with the laws of chemical affinity to the animal textures. As far as our researches have hitherto proceeded, the same law of chemical affinity holds good for the living as for the dead organism, without being in anywise influenced by the vital principle. In another point of view we meet with very important differences, according to whether the remedy has been introduced directly into the blood (by injection into the veins) or has been applied to the surfaces of the body, the skin, the mucous membrane of the bowels, &c. In the first instance the accidental decomposition is effected by the blood, which latter is in its turn altered. The thence-resulting new combinations, whether soluble or insoluble, mingle with the blood and circulate along with it through the body. If insoluble, they lodge in the capillaries, inducing local diseases, which do not belong to the proper effect of the medicine. If we consider in the other case this decomposition, the following phenomena present themselves: When applied to the stomach, rectum, or shin, we find the decomposition referrible to the reaction in the first place, and then to the secreting organ in the second. The changes which medicines undergo in this way are of the utmost importance, although little studied heretofore. Wherever the new combination is soluble, it is capable of absorption; but not so when insoluble." (p. 49.)

On comparing those different changes, we arrive at the following facts: 1. Certain constituents of the medicine remain unchanged and undissolved, and are neither decomposed nor enter into union with the animal textures. 2. Certain substances remain equally unchanged, but are fluid or soluble in the contents of the stomach, and therefore liable to absorption. 3. Many substances are not decomposed, but enter into combination with others, and are then absorbed. 4. Certain substances suffer, in obedience to the laws of chemistry, a partial decomposition. 5. Several medicinal agents become completely decomposed, and the elementary components unite differently, so as to form more complex combinations than before. The proofs of the absorption of medicines are based on the following circumstances:

"1. The presence of the remedy in the chyle and in the blood. 2. Its combinations with the animal solids. 3. Its separation by the excernent organs with the urine, the perspiration, the pulmonary exhalation, the milk, the saliva, &c. 4. Its disappearance from the first point of contact without being carried out of the body. 5. The supervention of a mere local effect, when the transit of the remedy into the blood by the part to which it has been applied is impossible. 6. The similarity of the phenomena in a remote part with those produced at the first point of contact. 7. The analogous effect produced by poisoned parts, and the poison itself." (p. 56.)

Several pages are occupied in establishing the truth of the above propositions, the author founding his arguments upon the experimental researches of Tiedemann, Gmelin, Wöhler, Müller, and others.

In discussing the effect of remedies in general, a clear explanation is given of the terms *action* and *reaction*, the sum of which constitutes the *effect*. Formerly, when much less was known than at present of the action of a remedy after its absorption, all the resulting symptoms were looked upon as *sympathetic phenomena*. Sympathy was, in short, the loophole through which writers on materia medica were glad of escaping, when hard pressed to explain the remote effects of remedial agents upon the system. Ignorance of sound physiology led them to this subterfuge. We are disposed to believe that the number of those agents which produce their whole effect, independently of absorption, is very small indeed. Thus hydrocyanic acid—which, applied in a concentrated form, appears to produce death more rapidly than it could be conveyed, by means of the circulation, from the capillaries to the brain, namely, in from half a minute to three minutes—has been brought forward as an instance of this kind. But even here the fact of sympathetic action is questionable; since Wedemeier ascertained that hydrocyanic acid, in a highly concentrated form, placed upon an exposed nerve, produced no general symptoms; and since we know that in from fifteen to thirty seconds the blood passes from one side of the body to the other.

Dr. Mitscherlich next goes on to describe the various channels by which medicines are introduced into the system; and then the different circumstances which tend to modify their action, as constitution, age, sex, habit, &c.

The changes effected upon the blood by medicinal substances are, according to our author, now pretty well understood.

"It has been already demonstrated that a small number of medicinal substances pass directly into the blood, altering its qualities; and the same thing can be indirectly proved to apply to most others. It now becomes a question, how do these particles which have been imbibed act upon this fluid? Do they form with it a homogeneous whole, or do they remain distinct from it, merely flowing in the same stream to the different parts and organs of the body? To elucidate this matter it is requisite to examine the state of the globules, and then that of the serum of the blood. . . . . . The globules of blood are partially dissolved by water, acetic acid, &c.; but these latter do not reach the blood in a simple form, but more or less changed; thus the water must previously have taken up several substances present in the stomach, and the acetic acid become associated with foreign matters, so that the action of these liquids upon the globules of blood by the medium of absorption is not the same as when brought into direct contact out of the body. The investigations relative to this subject upon the serum are still very defective; yet some facts of importance have been determined. After the continued employment of alkalies and their salts the coagulability of the fibrine and albumen of the blood is diminished, that vital fluid becoming clearer in colour, and more watery than before; and the inflammatory crust is essentially lessened, and at length disappears. As these salts have been detected in the blood, and as they alter its properties, both in the body and out of the body, it has been concluded that they operate through the medium of absorption, and that in consequence of the brightening of the colour, the globules at the same time undergo some change." (p. 103.)

The discovery of a medicament in an excretion is no proof of an increase of that excretion, as various colouring matters, as for example rhubarb, abundantly show. "In order to ascertain the real condition of an excretion," says Dr. Mitscherlich, "we must determine its specific gravity, the relative proportions of its solid and aqueous constituents, the nature and amount of saline and organic impregnation." His enquiries upon this head have led to some interesting results; namely, that in mercurial ptyalism the solid matters, which are greatly deficient, consist chiefly of salts; and that the urine, when copiously furnished after the ingestion of alkalies, has a lower specific gravity than natural, and is also wanting in solid contents.

It is well known that certain medicinal agents act with preference, so to speak, upon particular textures; and it has likewise been observed that the operation of some remedies is more specially directed to the function of one or more organs. Bitters improve the digestion, alkaline salts, cantharides, and the like, augment the discharge of urine, and narcotics affect the sensibility of the brain and spinal marrow.

It has been commonly supposed that there exists a class of medicines capable of exercising a direct influence upon the lymphatic system, in virtue of which solid deposits occurring in the structure of an organ or part are taken up and conveyed into the current of the circulation, without undergoing any essential change. There is, however, no conclusive evidence of the fact; and, as our author remarks, "it is much more probable that the deposits in question are first of all dissolved, the medicines acting on the blood in such a way as to fit it for this purpose, after which the absorption takes place in concurrence with an augmentation of certain secretions, as, for instance, that of the urine." (p. 108.)

In investigating the general effects of remedies it will be found that these correspond with their chemical and physical properties, and that this applies with equal force to the living as to the dead organism. Life interferes with neither the chemical affinities nor physical powers of bodies. It is true, indeed, that there are many phenomena in therapeutics which cannot be explained in this way, and have, accordingly, been viewed as the sequence of some unknown, or, to use the German term, *dynamical* action.

The systematic arrangement adopted by our author is based on the physiological and therapeutic effects taken conjointly; and for these reasons:

"On examining the effects of medicinal agents, we perceive an intimate connexion subsisting betwixt the physiological and the therapeutic; the only difference with regard to the latter is that the organism during disease is more or less modified, and the symptoms of reaction vary accordingly. In all cases where the nature and not merely the prominent symptoms of a disorder is understood, we may with more assurance reason as to the effect of a particular remedy, and frequently infer from the therapeutical effect the physiological, and conversely. On this ground we may fairly employ the physiological and therapeutical effects as points of division. When the nature of the disease eludes our apprehension, we may often discover it by reference to the physiological effect of such remedies as have proved effectual in combating or subduing it. Indeed, the two are so closely connected that, in contriving a rational arrangement of the materia medica, both the physiological and therapeutical effect ought to be taken into account." (p. 115.)

The first class comprehends Tonics; the second, Emollients; the third, Excitants; the fourth, Acrids; the fifth, Temperants; the sixth, Solvents; the seventh, Narcotics; the eighth, Alteratives; and the ninth, *Medicamina incertæ sedis.*

We sincerely hope that the subsequent parts of this work will be exempt from that taint of Galenism which pervades, to a greater or less extent most of the German treatises on the Materia Medica.

II. We have perused, with much satisfaction, the volume of Mr. Pereira. In addition to a good deal of original matter, the author has embodied a mass of valuable information, drawn from the most authentic sources, both foreign and British; and has displayed throughout a degree of labour and research, which deserves the highest commendation.

The first hundred pages, nearly, contain the general elements of pharmacology and therapeutics, comprehending the means of ascertaining the operation of medicines, their mode of action, their physiological effects, their absorption, their therapeutical effects, the parts of the body to which they are applied, and their classification. The author then proceeds to special pharmacology, adopting the natural-historical arrangement, "believing it to be the most convenient, and, on the whole, the least objectionable," (Preface, p. 1;) and at the same time pointing attention to the distinguishing characters of organized and inorganized beings, the peculiarities of chemical composition, of form and structure, of actions or functions.

The different remedies belonging to the inorganic kingdom occupy the remainder of the volume. Each of these is fully and copiously treated of under the following heads: General History; Natural History; Preparation; Properties; Characteristics and Composition; Physiological Effects on Vegetables, on Animals generally, on Man; Modus Operandi; Uses; Administration; Antidotes, when poisonous. Complex chemical decompositions are elucidated by appropriate diagrams; and, wherever the subject requires it, we find ample instructions relative to the applications of toxicology.

A work like the present, being itself a condensed summary of the subjects of which it treats, is, of course, unsusceptible of analysis; in noticing it we must, therefore, content ourselves with selecting, here and there, as we turn over its well-filled pages, a few passages that attract particular attention from their importance or peculiarity, or because they seem to claim from us some elucidation or qualification. The book is neither to be analyzed by the critic nor read by the buyer: it must lie for habitual reference on the table of the study.

"Every practitioner is familiar with a stomach complaint in which pain of a spasmodic character is the leading symptom, but which is not essentially accompanied by pyrexia, as in gastritis—by tendency to faint, as in cardialgia—by indigestion, as in dyspepsia, nor by loss of appetite; though one or more of these conditions may attend it. By some nosologists (as Sauvages and Sagar) it has been regarded as a distinct disease, and has been termed *gastrodynia*. It is not unfrequently accompanied by vomiting and precordial tenderness, which, however, cannot be regarded as indicative of inflammation, for various reasons; one of which is the alleviation of it often obtained by the use of stimulants and antispasmodics. What may be the precise pathological condition of this malady, I know not. Dr. Barlow (*Cyclopædia of Practical Medicine*, Art. *Gastrodynia*,) thinks the primary disease to be irritation or excitement of the mucous membrane of the stomach, whereby a redundant, dense, membranous, and opaque mucus is secreted, which accumulates and oppresses the stomach. The pain he supposes to arise from a contractile effort of the stomach to detach and expel the offending matter; but the immediate and permanent relief sometimes obtained by the use of hydrocyanic acid, is, I conceive, almost fatal to this hypothesis. Some time since, I prescribed the acid for a lady who had suffered for months with gastrodynia, and who was persuaded from her sensations, she had some organic disease. The remedy acted in the most surprising manner; in a few hours, to the astonishment of herself and friends, she was apparently quite well, and has since had no return of her complaint. It can hardly be imagined that irritation of stomach can be rapidly removed by a substance which is itself an irritant. For my own part, I conceive the affection to be, essentially, a disordered condition of the nerves supplying the stomach, or of the nervous centres from whence those nerves are derived; and that it is frequently, but not invariably, accompanied with the irritation of stomach alluded to by Dr. Barlow. But be the proximate cause of the disease what it may, the beneficial effects of the hydrocyanic acid, in some instances of gastrodynia, are most astonishing, while in others it totally fails. In all the cases in which I have tried it, I have obtained either perfect success or complete failure; I have met with no cases of partial relief. It not only allays pain, but relieves vomiting, and in the latter cases, frequently when all other remedies fail. . . .

"I have also found it useful in a painful affection of the bowels analogous to that of the stomach, and which, therefore, might with propriety be termed *enterodynia*. The most remarkable case of this kind which I have met with, was that of a gentleman, a relative of one of my pupils. He had suffered for several months excruciating pain in the bowels, commencing daily about two o'clock, and only ceasing at night. It was apparently a consequence of an ague. He had been under the care of several country practitioners, and had tried a number of remedies (including opium and sulphate of quinia) without the least benefit. I advised the employment of the hydrocyanic acid, and accordingly five minims were administered at the commencement of a paroxysm: the remedy acted like a charm; all the unpleasant symptoms immediately disappeared. Several doses of the acid were given before the time of the succeeding paroxysm, but the disease never returned; and after employing the acid for a few days longer, he went back to the country completely cured.

"I have seen hydrocyanic acid used with great success to allay vomiting and purging in severe forms of the ordinary English cholera, when opium has completely failed. In Asiatic or malignant cholera it has occasionally appeared to be serviceable.

"As a remedy for affections of the pulmonary organs, hydrocyanic acid was at one time in great repute. It was said to be capable of curing slight inflammation of the lungs, without the necessity of bloodletting, of suspending or curing incipient phthisis, while in confirmed cases it soothed the approach of death, of curing hooping-cough, and of removing all the symptoms of spasmodic asthma. (See *Dr. Granville's Treatise*, and also *Magendie's Recherches sur l'Emploi de l'Acide Prussique*, 1819.) Experience has shown the fallacy of most of these statements. I have employed hydrocyanic acid in a considerable number of cases of phthisis, and have occasionally fancied that it relieved the cough and night-sweats; but these effects were only temporary. Cases of genuine spasmodic asthma are rare; but in two instances in which I have seen the acid employed, no relief was obtained. In allaying cough, (especially the kind called spasmodic) I have on several occasions, found it useful; but it has so frequently disappointed my expectations, that I now rarely employ it in any pulmonary diseases. I have never observed any ill effects from its use in these cases, though others assert they have.

"It has been employed in affections of the nervous system. Cases of hysteria, epilepsy, chorea, and tetanus, have been published, in which this remedy has been found beneficial. I have seen it employed in the first three of these affections, but without any evident relief. It has been proposed and tried in hydrophobia; it apparently mitigated the symptoms. Dr. Hall (*Lect. on the Nerv. Syst.* p. 155,) proposes that, in addition to the use of this acid, tracheotomy, as suggested by Mr. Mayo, should be tried.

"Hydrocyanic acid has been administered as an anodyne in several painful affections, namely, cancer, tic-douloureux, rheumatism, &c., but, with a few exceptions, it has not been found serviceable." (p. 248.)

Page 261 is embellished with a small woodcut, representing the cascade of Vinagre, in Colombia, so called from its waters being impregnated with sulphuric and hydrochloric acids. The author might at the same time have mentioned the remarkable occurrence of *pure* sulphuric acid in large quantities, both in a diluted and concentrated state, in the town of Byron, Genessee county, ten miles south of the Erie canal. The place has been known in the vicinity for seventeen years, by the name of the *Sour Springs.* It has been described by Professor Eaton. (*Quart. Journ. of Science*, p. 200, 1829.)

The compounds of potassium are well described. In speaking of potash, the fact pointed out by Gay Lussac, of a great number of animal and vegetable substances when acted upon by it in the caustic form, being transformed into oxalic acid with a simultaneous production of acetic acid and water, has escaped notice. In the enumeration of the remedial uses of the *liquor potassæ*, we find the following judicious observations:

"The alkalies have been lately celebrated for producing beneficial effects in those *inflammations* which have a disposition to terminate in exudation and adhesion; that is to say, those that frequently give rise to the formation of false membranes or of adhesion; such for example, as croup, pleurisy, and peritonitis. If experience should subsequently confirm the assertions already made respecting their efficacy, we shall have another analogy between the operation of alkalies and of mercury. Theoretically, it has been argued, the alkalies are likely to be beneficial in these diseases on two accounts; first, they have a tendency to diminish the supposed plasticity of the blood, which some have assumed (though without proof) to be connected with the exudation; and, secondly, we find these albuminous deposits readily dissolve, out of the body, in alkaline liquids: but arguments of this kind are to be received with great caution. In conclusion, I may add that Eggert recommends the alkalies as specifics against croup, though Sundelin (*Heilmittel*, 1er Bd. § 182,) found them inoperative. Hellwag employed them to cause the removal of the deposited lymph: Memminger gave them with benefit in hooping-cough; Mascagni, in pleurisy and peripneumony. (*Vogt*, *Lehrbuch d. Pharmakodyn*, 2er Bd. p. 529.) It is asserted that in the latter complaints, the alkalies render the expectorated matter less viscid, and at the same time, act powerfully, as diaphoretics and duretics." (p. 279.)

The following remarks upon the exhibition of lime are interesting:

"When administered internally, it neutralizes the free acid of the gastric juice, diminishes the secretions of the gastro-intestinal membrane, and thereby occasions thirst and constipation. It frequently gives rise to uneasiness of stomach, disordered digestion, and not unfrequently to vomiting. After its absorption, it increases the secretion of urine, and diminishes the excessive formation or deposition of uric acid and the urates. With this exception, it does not, as the alkalies, promote the action of the different secreting organs; but on the other hand, diminishes it, and has been in consequence termed an astringent. But it does not possess the corrugating action of the astringent vegetables, or of many of the metallic salts: it is rather a drying remedy and might be more correctly termed a *desiccant* than astringent. In this respect, lime differs from the alkalies, but is analogous to the oxide of zinc. Vogt (*Pharmak.*) considers it to be intermediate between the two. Weichard and others have ascribed to lime an antispasmodic property; and if this be true, its relation to zinc is still further proved.

"A power of exciting and changing the mode of action of the absorbent vessels and glands has been ascribed to lime-water, and probably with foundation. At any rate, under the use of it, glandular enlargements have become softer and smaller. Sundelin (*Heilmittel*) says that the excessive use of lime does not, as in the case of the alkalies, bring about a scorbutic diathesis, but a general drying and constriction, analogous to that caused by zinc." (p. 344.)

Pages 374 to 397 contain a very excellent account of the arsenical preparations. The nascent hydrogen test of Marsh, for detecting arsenious acid, is fully explained and illustrated by appropriate engravings on wood. In pointing out the fallacy of this test, caused by antimonial impregnation, the author has omitted to indicate a ready means of obviating any fallacy whatever, derived from the property of the arseniuretted hydrogen gas of depositing its arsenic by heat. Hence, in order to render the apparatus at once sure and efficient, it is only requisite to conduct the gas, in proportion as it is disengaged, along a tube of glass kept at a red heat by the flame of a spirit-lamp. Where more precision is desired, a determinate quantity of copper reduced by hydrogen is to be placed in the ignited part of the tube. From the resulting arseniuret of a copper, characterized by its silvery whiteness, we can easily appreciate with extreme exactitude the amount of arsenic combined with hydrogen. A simple means of distinguishing arseniuretted hydrogen from antimoniuretted hydrogen has also escaped our author's notice; it consists in introducing a small portion of chlorine gas, which precipitates from the one metallic arsenic, while it leaves the other unchanged.

The precautions to be observed in administering this potent but dangerous remedy are given in detail at pages 393 and 395. A fact stated at p. 386 ought never to be forgotten by the medical practitioner, namely, "if the use of small doses of arsenious acid be continued for a long period, it acts as a slow poison; and if persevered in, will ultimately occasion death. The same effects take place, in a shorter period, from the administration of large medicinal doses. Sometimes the digestive apparatus, at other times the nervous system, first shows symptoms of the poisonous operation of this agent."

Among the antidotes in cases of poisoning by arsenic, the hydrated sesquioxide of iron has been lately brought into repute; and a very complete account of its effects will be found in a former Number of this Review.* As however its efficacy as an antidote has been denied, (*London Med. Gazette*, xv. p. 220,) we think it right to put our readers in possession of certain particulars in regard to its preparation, which MM. Bunsen and Berthold hold to be essential to its successful operation.† In order to obtain the hydrated sesquioxide in a state of purity, we are to peroxidize by nitric acid, with the aid of heat, a solution of pure sulphate of iron, then add caustic ammonia in excess, and wash the precipitate by decantation. It is important that the sulphate be quite dissolved before the nitric acid be added in small portions, otherwise there separates a considerable quantity of the neutral sulphate of ses-

* Vol. IV. p. 237. † Archiv der Pharm., Bd. xi. Cah. iii. p. 323.

quioxide, which falls to the bottom in the form of a yellowish powder, of very sparing solubility. The muriate of iron is ill adapted for the preparation of this oxide, because in precipitating with ammonia we run the risk of obtaining in combination a large amount of the basic chloride.

In order not to remove the water from the precipitated hydrate of sesquioxide, so as to diminish in the slightest its feeble state of aggregation, it should not be filtered; but after settling for some hours the supernatant liquor is to be decanted, and the residuum preserved under water in well-closed vessels.

In alluding to the diseases in which nitrate of silver has been given internally with advantage, such as epilepsy, chorea, angina pectoris, chronic affections of the stomach, &c., Mr. Pereira says (p. 430) "its *methodus medendi* is inexplicable." We are disposed to concur with the ingenious explanation suggested by Dr. Osborne, that in all the above cases nitrate of silver allays irritation of the gastric membrane, and does good primarily in that way. (*Dublin Journal of Med. Science*, Jan. 1839.)

Mercury and its compounds are discussed at great length and in a very masterly manner. The author's summary of their morbid effects is admirable. (pp. 441-3.) In reference to Dr. Wilson Philip's notion, that "mercury has a specific operation on the liver, a power not merely of exciting its functions, but of correcting the various derangements of that function in a way which it does not possess with respect to any other organ, and which no other medicine possesses with respect to the liver;"* he very justly confesses his ignorance of any facts warranting the assertion. (p. 445.)

At page 461 binoxide of mercury is declared to be "insoluble in water," whether procured by calcination or precipitation. It appears, however, from some recent experiments of M. Bondet, recorded in a late Number of the Journal de Pharmacie, that perfectly pure binoxide of mercury is slightly soluble in distilled water.

In indicating the different substances which decompose corrosive sublimate, the author might have annexed the mucilage of quince-seed and that of salop, which act instantaneously; a fact first noted by M. Fabian, (see *Quart. Journ. of Science for* 1830, p. 198.)

In the history of zinc (p. 521) it is stated that, "although the ancients were acquainted with the method of converting copper into brass by means of an ore of zinc, yet they were unacquainted with metallic zinc, one of the constituents of this alloy. Albertus Magnus, who died in 1280, is the first who expressly mentions this metal." But a passage in Strabo authorizes the belief that they also knew this metal in its separate state. The geographer says† that near Andeira, a town of Troas, is found a stone, which being burnt becomes iron; and distils false silver, (ἀποσταζει ψευδαργυρον,) when heated together with a certain earth, which, receiving the addition of copper, forms the alloy that some call brass (ὀρειχαλκον). He adds, respecting this *false silver*, which was probably our zinc, that it occurs also near the Tmolus. Stephanus states the same thing in somewhat clearer words, and refers to both Theopompus and Strabo as authorities.‡

* On the Influence of Minute Doses of Mercury, p. 14.

† Strabo, p. 610.

‡ Steph. de Urbibus, word Andeira.

In treating of the salts of zinc, it is observed of the acetate that "its effects are analogous to, though milder than the sulphate of zinc, but more energetic than the oxide." (p. 529.) Its dose, when exhibited internally as a tonic or antispasmodic, is one or two grains gradually increased. As an emetic, the dose is five or ten grains. Whereas the dose of the sulphate, as an emetic, is stated to be from ten to twenty grains; as a *tonic*, *antispasmodic*, or *expectorant*, from one to five grains. Does not this involve a posological contradiction?

Mr. Pereira constantly uses the term hydrosulphuric acid as synonymous with sulphuretted hydrogen. This we conceive objectionable, inasmuch as hydrosulphuric acid signified in Sir H. Davy's nomenclature hydrous sulphuric acid.

In concluding our notice of this the first part of Mr. Pereira's work, we have to express our thorough conviction of its intrinsic worth and usefulness. It contains a most clear and comprehensive exposition of the principles and practice of therapeutics; and, when complete, will constitute a very valuable addition to the literature of the materia medica. Perhaps, the only objectionable part are the woodcuts, many being commonplace, and others ill adapted to convey a distinct notion of the process they are intended to illustrate. The whole style of the engraving is, indeed, below the level of the present condition of the art, and calls for improvement in the next edition of the book.

III. As the more important parts of Dr. Dierbach's compilation have been embodied in the work of Mr. Pereira, we shall content ourselves with furnishing our readers with a brief analysis. Pages 1-49 contain the modern literature of the materia medica under the following heads: the knowledge of drugs, medical mineralogy, medical botany, medical zoology, dietetics, the doctrine of the effects of remedies, toxicology, the art of prescribing, pharmacopœias, taxation of drugs. The next eleven sections (pp. 50-648) are occupied in considering remedies individually. I. Plants or parts of plants which have been recommended within the last few years, (pp. 50-225, secs. 1-102:) those growing in Europe, wild or cultivated, occupy secs. 1-54: whilst the exotic vegetable productions occupy secs. 55-102. II. New preparations of vegetable substances, (pp. 225-312, secs. 103-146:) and 1, Mild nutritious, or tonic excitant articles, for the most part bitter, secs. 103-121; 2, Articles more or less acrid, determining occasionally violent vomiting or purging, secs. 122-131; 3, Potent, chiefly narcotic drugs, secs. 132-142; 4, New remedies allied to the acids, secs. 143-146. III. New preparations procured by the combustion or the dry distillation of organic substances, (pp. 313-343, secs. 147-156.) IV. Various animal products, and the resulting preparations, (pp. 343-358, secs. 157-166.) V. Hydrocyanic acid and other compounds of cyanogen (pp. 358-388, secs. 197-176.) VI. Chlorine, iodine, bromine, and their respective preparations, (pp. 388-463, secs. 177-205.) VII. Sulphur and sulphuretted remedies, (pp. 463-472, secs. 206-210.) VIII. Salts, soap, metallic preparations, (pp. 473-542, secs. 211-236.) IX. New methods of employing certain gases, (pp. 543-555, secs. 237-243.) X. Oil of grain and hydrogenated oil of grain, (Germ. *Fermentoyl*,) (pp. 555-558, sec. 244.) xi. Pharmacological miscellanies, (pp. 558-648, secs. 245-253.) At the beginning of the work there is an index of the remedies according to their therapeutic effects. Dr. Dierbach, actuated by the true spirit of German industry, may be said to have supplied a register of all the most recent discoveries in the materia medica, which cannot fail to be of use to the medical practitioner and student of pharmacy; containing, as it does, a full and copious account of the nature and properties of the several articles of the diseases in which they have been found useful, of their doses and forms of employment.

IV. Dr. Forcke's lucubrations will not detain us long. They are evidently those of a tyro, dazzled by accounts of the virtues of a remedy, which subsequent experience has proved to be in many instances exaggerated, in others false. The following is his account of the effects of veratria upon the human body in the healthy and morbid conditions.

"After a person has taken, for two or three times, from one sixth to one fourth of a grain of veratria, frequently, indeed, in from half an hour to an hour after the first dose, there arises a feeling of tingling, sparkling, prickling, (gefühl von prickeln, funkeln, oder pinkeln,) in parts remote from the stomach, most commonly in the points of the fingers and toes, sometimes in the elbows, bends of the knees, and shoulders, occasionally on the forehead or over the eyebrows, more rarely and at a longer interval in the thighs, abdomen, or back. Simultaneous with this, or soon after, some patients experience a sense of warmth, others of cold referred to different regions of the extremities and trunk, mostly in the hands and soles of the feet, the knees, and mouth. While one has the feeling as if a stream of warm air or drops of hot water were issuing from these parts, another perceives, as it were, a frozen atmosphere around the feet and particularly the knees, or as if cold water was poured upon them. Some compare the sensation in the mouth to that produced by sucking peppermint. In general, the warmth occurs where there is integrity of the vital powers but abdominal torpor, while old hypochondriacs and hysterical women, with predominant asthenia, experience the cold. . . . . Besides the above sensations, there is occasionally superinduced this peculiar phenomenon, namely, that a pain, which may have been of long standing in some part of the body, either suddenly disappears altogether, or else is replaced by another, equally sudden in its invasion, in some distant part. Examples are not wanting of a limb or of the muscles of the face, especially if previously the subject of painful or spasmodic paroxysms, becoming seized with starting and tremor soon after the taking of the medicine. Thus, in from half an hour to an hour after the dose had been swallowed, parts began to start, which were in a half paralyzed state from apoplexy, or which used to present violent spasms or trembling during severe paroxysms of *tic douloureux*. In some cases the development of heat was very marked. In the case of a torpid lymphatic subject, in whom, from "abdominal epilepsy," the left arm had got into a half-palsied state, attended with frequent twitchings, a fleeting but sensible warmth succeeded the application of the veratrine, which had never been noticed at any other time. A few doses sufficed to restore permanent warmth to the feet of a feeble, emaciated, melancholy female, which for years before had remained of an icy coldness. This seems to show that the remedy acts powerfully upon the organic nerves." (pp. 20-2.)

The author is of opinion that all the cases of facial neuralgia which have yielded to the veratria, have been merely dependent on sensory and functional disorders of the nerves. (p. 46.) On this point he has our full concurrence. After adducing a few instances where benefit seems to have been derived from the employment of this alkaloid in neuralgia, partial paralysis, and diseased heart; others are brought forward of hooping-cough catalepsy, epilepsy, eclyse (fainting), hypochondriasis, hysteria, dropsy, &c., in which the results are by no means satisfactory.

V. Dr. Köchlin, a follower of Paracelsus, and a humoral pathologist, has favoured the profession with a treatise on the healing virtues of metals. His doses are minute, for without being a homœopath, he restricts them to the 100th of a grain. After some preliminary observations on sensibility, irritability, and the like, the author proceeds to discuss the action of metallic substances upon the organism. This, in some instances, is that of expansion, in others that of contraction. He informs us that some remedies are *mediate*, others *immediate* in their effects. Thus tannin and heat illustrate the latter, while whatever changes the *crasis* and *plasticity* of the blood pertain to the former. Iron and mercury serve as instances; the first augmenting, and the second diminishing the *plasticity* of that fluid; but they are not the only metals endued with such powers. He next passes on to the liquefacient (*verflüssigenden*) resolvent, and then to the antiphlogistic action of metallic substances; and repudiates the notion entertained by some authors of there being a specific qualitative and a specific local action.

The different external influences and conditions which concur towards the preservation of life, and have to be taken into account in the employment of remedies, are fully noticed. As peculiarities, we may remark, that the author lays great stress on the necessity and importance of selecting the preparation best suited for accomplishing the end in view, considering as most efficient the union of the metallic hydrochlorates with sal-ammoniac, and urges the propriety of the medicine being swallowed directly after taking food. In the subsequent part of the book he treats, 1, of the action of metals upon the organism generally; 2, of their therapeutic agency; and 3, of the virtues of the cupro-ammoniacal solution, dignified by the title of the *Liquor of Köchlin*, and other cupreous preparations in particular. A series of extracts culled from German journals, and letters from German doctors, forming an appendix of twenty-eight pages, attest the wonderful powers of the pet liquor. Vogt, in his Pharmakodynamik (Bd. i. p. 320), informs us, that Köchlin having learned that a Dr. Beisser in the East Indies possessed a nostrum of a cupreous nature, which proved remedial in certain instances of syphilis, where mecurials failed, proposed his liquor as a substitute. The same eminent writer states that it has answered in the hands of other practitioners, and is highly serviceable in inveterate atonic syphilis, in old eroding scrofula and rachitic ulcers, in the so-called *dyscrasia ulcerosa*, in cancerous sores, and *esthiomenous* affections of the skin. Kopp recommends its use in certain abdominal disorders, in *infarctus* and *physconia*, in incipient mesenteric disease, and especially in pyrosis with cramp of the stomach. It does not, like other cupreous preparations of equivalent strength, produce gastric derangement. The solution is prepared as follows: take of copper-filings one drachm, of aqueous ammonia an ounce and a half, digest in the cold, until the liquid assume a blue colour; to an ounce of this add two drachms and forty-two drops of muriatic acid, and dilute the resulting combination with six pounds and a half and two drachms of distilled water. The dose for a child is a teaspoonful.

We deem it right to state that we consider the above formula to be faulty, inasmuch as the proportion of copper held in solution will vary according to circumstances. The blue tint depends wholly upon the absorption of oxygen; caustic ammonia may be kept in contact with copper-filings for an indefinite period without changing colour, provided the atmospheric air be excluded. It is only by a process of alternate coloration and decoloration, that is to say, by an alternate reproduction of the protoxide and dioxide that the ammonia becomes fully saturated.

---

